# 1430. Descriptive Epidemiology of Emergency Department Visits with cUTI in the US, 2012-2018

**DOI:** 10.1093/ofid/ofab466.1622

**Published:** 2021-12-04

**Authors:** Marya Zilberberg, Brian Nathanson, Kate Sulham, Andrew F Shorr

**Affiliations:** 1 EviMed Research Group, LLC, Goshen, MA; 2 OptiStatim, LLC, Longmeadow, MA; 3 Spero Therapeutics, Cambridge, MA; 4 Medstar Washington Hospital Center, Washington, DC

## Abstract

**Background:**

Urinary tract infections (UTI) represent a substantial burden to the healthcare system. In the early 2000s annual UTI admissions numbered 100,000, and these infections resulted in over 1 million emergency department (ED) visits. While only a fraction of total UTI volume, the estimated cost of complicated (cUTI) to the healthcare system exceeded &3.5 billion. We set out to evaluate the contemporary burden of cUTI in the US in terms of ED visits annually.

**Methods:**

We conducted a retrospective multicenter cohort study within the National Emergency Department (NEDS) database, a 20-percent stratified sample of all US hospital-based EDs, from 2012-2018, to explore characteristics of patients discharged with a cUTI diagnosis. We applied a previously published algorithm to identify cUTI using administrative coding. We applied survey methods to develop national estimates.

**Results:**

Among 3,010,997 ED visits with cUTI, 43.3% were female, and 59.0% were age 65 years or older. Commensurately, Medicare was the primary payor in 62.8% of the visits. The majority of the patients (59.1%) presented to metropolitan teaching hospitals, and plurality were in the Southern US (39.6%). There was a narrow range in the visits’ seasonal variation, from 6.4% occurring in February to 7.9% in October. cUTI was the principal diagnosis in 48.5% of all cUTI visits. In the remaining 51.5%, sepsis was the most common principal diagnosis (33.9%), but severe sepsis and septic shock codes each appeared in 4.9%. Of all cUTI ED visits, 21.4% had catheter-associated UTI. While only 19.8% had a code for pyelonephritis, 2,050,548 (68.1%) were admitted to the hospital. Mortality in the ED was 0.02%.

**Conclusion:**

During the seven-year span, there were over 3 million ED visits for cUTI. Although fewer than 1 in 10 patients met criteria for severe sepsis/septic shock, approximately 2/3rds of cUTI patients presenting to the ED were subsequently hospitalized.

**Disclosures:**

**Marya Zilberberg, MD, MPH**, **Cleveland Clinic** (Consultant)**J&J** (Shareholder)**Lungpacer** (Consultant, Grant/Research Support)**Merck** (Grant/Research Support)**scPharma** (Consultant)**Sedana** (Consultant, Grant/Research Support)**Spero** (Grant/Research Support) **Brian Nathanson, PhD**, **Lungpacer** (Grant/Research Support)**Merck** (Grant/Research Support)**Spero** (Grant/Research Support) **Kate Sulham, MPH**, **Spero Therapeutics** (Consultant) **Andrew F. Shorr, MD, MPH, MBA**, **Merck** (Consultant)

